# Single-cell transcriptomes reveal molecular specializations of neuronal cell types in the developing cerebellum

**DOI:** 10.1093/jmcb/mjy089

**Published:** 2019-01-25

**Authors:** Jian Peng, Ai-li Sheng, Qi Xiao, Libing Shen, Xiang-Chun Ju, Min Zhang, Si-Ting He, Chao Wu, Zhen-Ge Luo

**Affiliations:** 1 Institute of Neuroscience, State Key Laboratory of Neuroscience, Center for Excellence in Brain Science and Intelligence Technology, Chinese Academy of Sciences, Shanghai, China; 2 University of Chinese Academy of Sciences, Beijing, China; 3 ShanghaiTech University, Shanghai, China; 4 Co-innovation Center of Neuroregeneration, Nantong University, Nantong, China

## Abstract

The cerebellum is critical for controlling motor and non-motor functions via cerebellar circuit that is composed of defined cell types, which approximately account for more than half of neurons in mammals. The molecular mechanisms controlling developmental progression and maturation processes of various cerebellar cell types need systematic investigation. Here, we analyzed transcriptome profiles of 21119 single cells of the postnatal mouse cerebellum and identified eight main cell clusters. Functional annotation of differentially expressed genes revealed trajectory hierarchies of granule cells (GCs) at various states and implied roles of mitochondrion and ATPases in the maturation of Purkinje cells (PCs), the sole output cells of the cerebellar cortex. Furthermore, we analyzed gene expression patterns and co-expression networks of 28 ataxia risk genes, and found that most of them are related with biological process of mitochondrion and around half of them are enriched in PCs. Our results also suggested core transcription factors that are correlated with interneuron differentiation and characteristics for the expression of secretory proteins in glia cells, which may participate in neuronal modulation. Thus, this study presents a systematic landscape of cerebellar gene expression in defined cell types and a general gene expression framework for cerebellar development and dysfunction.

## Introduction

The cerebellum is known for its critical roles in motor coordination, posture balance, and motor learning (Glickstein et al., 2009; Caligiore et al., 2017; Lang et al., 2017). Recently, accumulating lines of evidence have suggested its roles in non-motor functions such as cognition and emotion (Koziol et al., 2014; Baumann et al., 2015). These diverse functions are thought to be encoded by cerebellar circuits that are composed of relatively few cell types distributed in the cerebellar cortex and cerebellar nuclei (Buckner, 2013), such as GABAergic Purkinje cells (PCs) with an elaborate dendritic arborization extending into the superficial molecular layer, glutamatergic excitatory granule cells (GCs) in the innermost granular layer, and several types of interneurons (Marzban et al., 2014; Lackey et al., 2018). Although microscopic anatomy and canonical microcircuit of the cerebellar cortex have been extensively studied, the molecular mechanisms governing cerebellar neuronal fate determination and maturation remain unclear.

The cerebellar cytostructure emerges during early embryonic stage from multiple germinal zones, mainly ventricular zone (VZ) of the forth ventricle wall and the rhombic lip (RL) (Hatten and Heintz, 1995), and migrate to distinct layers via tangential or radial migratory pathways. PCs, the output of cerebellar cortex, are derived from the VZ of mouse at E10-E13 (Wang and Zoghbi, 2001), followed by radial migration to form a plate of several cell layers and then a monolayer configuration after birth (Marzban et al., 2014). Then, PCs experience sequential cell shape changes, from a distinct bipolar morphology to a highly elaborated dendritic configuration that is flattened within the sagittal plane (Sotelo and Rossi, 2013). The cerebellar GCs, the most abundant neurons in the vertebrate brain, are derived from the RL in mice at E12.5-17.5, followed by tangentially migration to form the external germinal layer (EGL), a temporary population of proliferating cells. At birth, some granule precursors (GPs) in inner EGL exit the cell cycle and differentiate into mature GCs resulting in the gradual disappearance of the EGL. Subsequently, postmitotic GCs exhibit dynamic morphological changes with leading processes guiding the inward migration along the radial fibers of the Bergmann glia to populate at the internal granular layer (IGL) (Rakic, 1971; Adams et al., 2002), and this process becomes obvious at P5 and is completed by P20 (Altman and Bayer, 1997). At IGL, GCs project an ascending axon that bifurcates at the molecular layer to form the parallel fibers, which synapse with dendrites of PCs or interneurons (Komuro and Yacubova, 2003). The cerebellar interneurons, including stellate and basket cells in the molecular layer, candelabrum cells in the PC layer, and Golgi cells in the granular layer, are born postnatally from arguably diverse origins (Hoshino, 2006). Although many genes and signaling pathways have been shown to be involved in the development, fate determination, and migration of cerebellar neurons, many questions remain unclear. For example, how molecular cascades finely tune neurogenesis and migration of GCs, what genetic factors specify characteristic morphology of PCs, and which factors determine specific synaptic connections.

Cerebellar abnormality and dysfunction cause a number of neurological and neuropsychiatric symptoms, such as ataxia, tremor, autism spectrum disorder, and schizophrenia (Bastian, 2011; D'Angelo and Casali, 2012; Reeber et al., 2013). Understanding the cell-type specific expression of disease-related genes may help understand circuitry basis of these diseases and design specific interventions. High throughput single-cell RNA sequencing (scRNA-seq) allows for deeper understanding of molecular specification of various cell types in distinct brain regions of given species. Recently, several studies have presented transcriptional profiles of the developmental murine cerebellum (Carter et al., 2018; Gupta et al., 2018; Rosenberg et al., 2018). However, their analyses focused on the expression of transcription factors (TFs) associated with fate determination of GCs or glutamatergic lineages.

Here, we performed the droplet-based scRNA-seq to survey the cerebellar cell types and identified gene expression profiling of the murine cerebellum at postnatal days 0 and 8. We show temporally dynamic gene expression patterns correlated with states of various neuronal cell types, including GCs and GPs, developing PCs, as well as interneurons and glia cells. This analysis also led to identification of novel markers of various cerebellar cell types and suggested factors that might be involved in neuronal differentiation, morphogenesis, or neuro-glia interactions. Moreover, enriched expression of ataxia-related genes in PCs indicates cellular mechanisms of cerebellum-associated diseases.

## Results

### Cell types of the developing cerebellum

**Figure 1:**
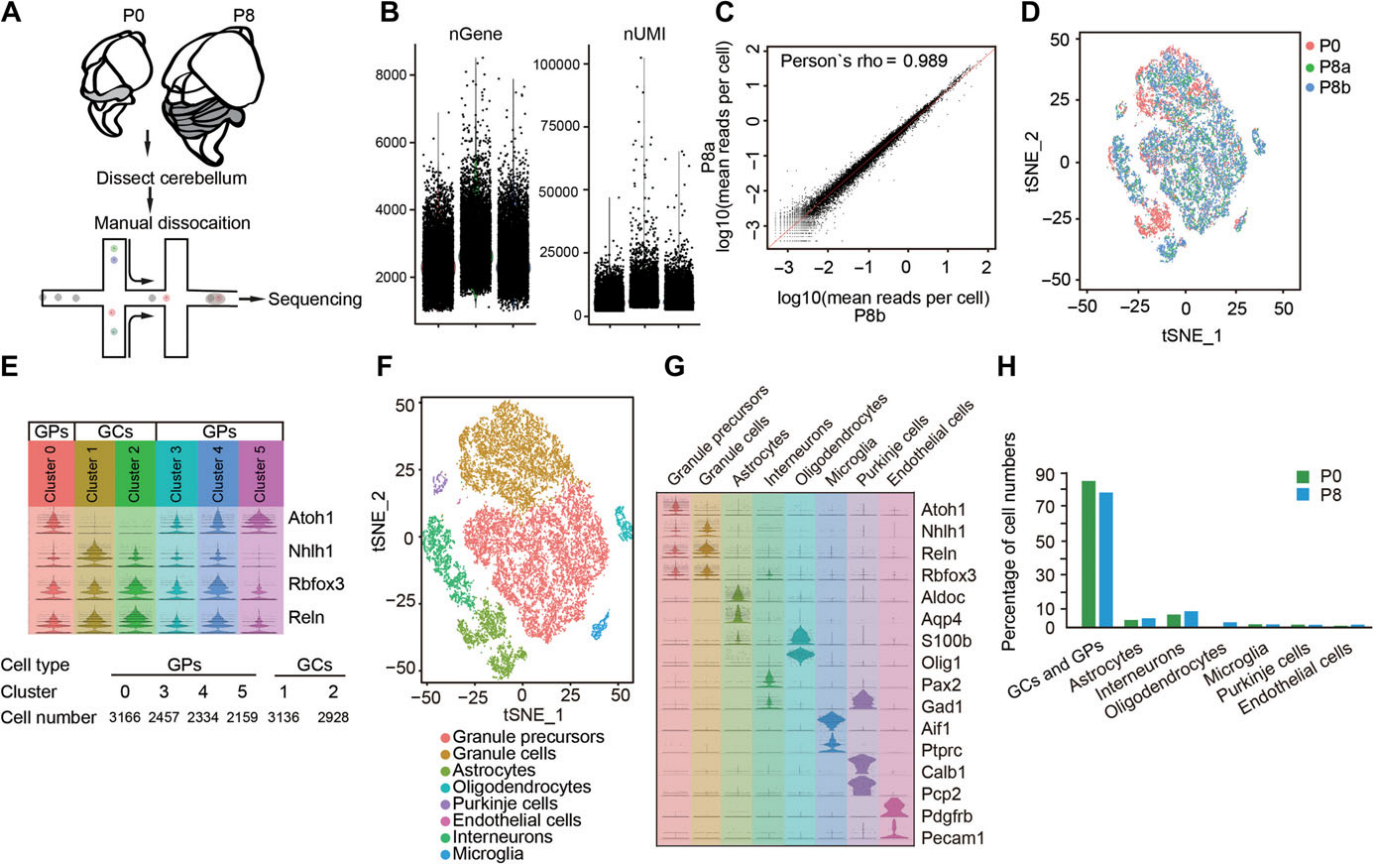
Cell type classification in the mouse cerebellum. (**A**) Schematic representation of the experimental processes consisting of cerebellum dissection, cell dissociation and droplet-based RNA-seq analysis. (**B**) The number of genes (nGene) and UMIs (nUMI) identified in each sample (1 from P0 and 2 from P8). (**C**) Gene expression reads are highly correlated between two P8 samples. (**D**) Two-dimensional t-SNE distribution of cell clusters in three samples. Note the large overlap between two P8 samples but not between P0 and P8 samples. (**E**) Identification of granule cells (GCs) or precursors (GPs) based on the expression of known genes. (**F**) t-SNE map showing annotation of indicated cerebellar cell clusters. (**G**) Identification of cerebellar cell types based on the expression of known linage-specific genes. (**H**) Percentage of cell types in the cerebellum of P0 and P8 mice.

To investigate the postnatal development of the cerebellum, we performed scRNA-seq analysis for samples from postnatal day 0 and 8 using the droplet system from 10x Genomics Chromium (Macosko et al., 2015; Zilionis et al., 2017). After filtering out outliers with extremely low (<1000) and high (>6500) gene numbers, which represented probable dead cells and duplicates, respectively, a total of 21119 single cells from one P0 sample and two independent P8 samples were analyzed (Figure 1A and Supplementary Dataset S1). A means of 58994 unique molecule identifiers (UMIs) and a median of 2615 genes were detected in each cell (Figure 1B and Supplementary Figure S1A–C). To examine batch effect and individual variance, we performed Pearson correlation coefficients and canonical correlation analysis (CCA) of three samples (Butler et al., 2018). As shown in Figure 1C, the mean reads per cell of two P8 samples were highly correlated (*r* = 0.989). The cell distribution from two P8 samples was tightly close as visualized in 2D t-distributed stochastic neighbor embedding (t-SNE) plot (Figure 1D), suggesting the reproducibility of our data collection. To classify major cell types in the developing cerebellum, we performed a multi-set canonical correlation analysis (Multi-CCA) on gene expression matrix and integrated three datasets (one P0 and two P8) into a single one, and identified shared correlation structures (canonical correction vectors, CC) across datasets (Butler et al., 2018). Because we have begun to see drop-off in signals of shared correction strength before CC 15, we chose CC 1 to 15 for subsequent analysis and t-SNE presentation (Supplementary Figure S1D). We identified 12 transcriptionally distinct cell clusters using Seurat R package (Satija et al., 2015). Then, we used a series of known cerebellar cell lineage markers to annotate each cluster, such as *Atoh1* marking Clusters 0, 3, 4, and 5 as GPs and *Rbfox3* marking Clusters 1 and 2 as GCs (Ben-Arie et al., 1997; Weyer and Schilling, 2003) (Figure 1E). Notably, *Rbfox3* is also expressed in a fraction of *Atoh1*-positive GPs, suggesting intermediate state of the population. Other clusters represent astrocytes, interneurons, oligodendrocytes, microglia, PCs and endothelial cells (Figure 1F and G; Supplementary Figure S2A, B and Dataset S1). Our analysis did not identify a separate Bergmann glial cell population, probably because astrocytes and Bergmann glial cells are originated from common precursors (Fleming et al., 2013). Indeed, we found that astrocytes expressed Bergmann glial cell marker genes, such as *Sox1* and *Sox9* (Supplementary Figure S2C). As a type of GABAergic neuron, PCs expressed *Gad1*, in addition to known specific marker genes encoding *Calbindin 1* (*Calb1*) and *Purkinje cell protein 2* (*Pcp2*) (Figure 1G). In line with the notion that cerebellar GCs is the biggest cell population in the vertebrate brain, GPs and GCs accounted for 78% of total cells at P8, whereas PCs accounted for only 1.43% (Figure 1H and Supplementary Dataset S2). Although different cell types are distinct in the size of cell body, the numbers of genes and UMIs of each cell type were similar (Supplementary Figure S3A and B). Notably, the percentage of mitochondrial transcript reads in PCs was bigger than that in other cell types (Supplementary Figure S3C), suggesting that PCs may contain more mitochondria.

### Transcriptional profile of developing PCs

**Figure 2:**
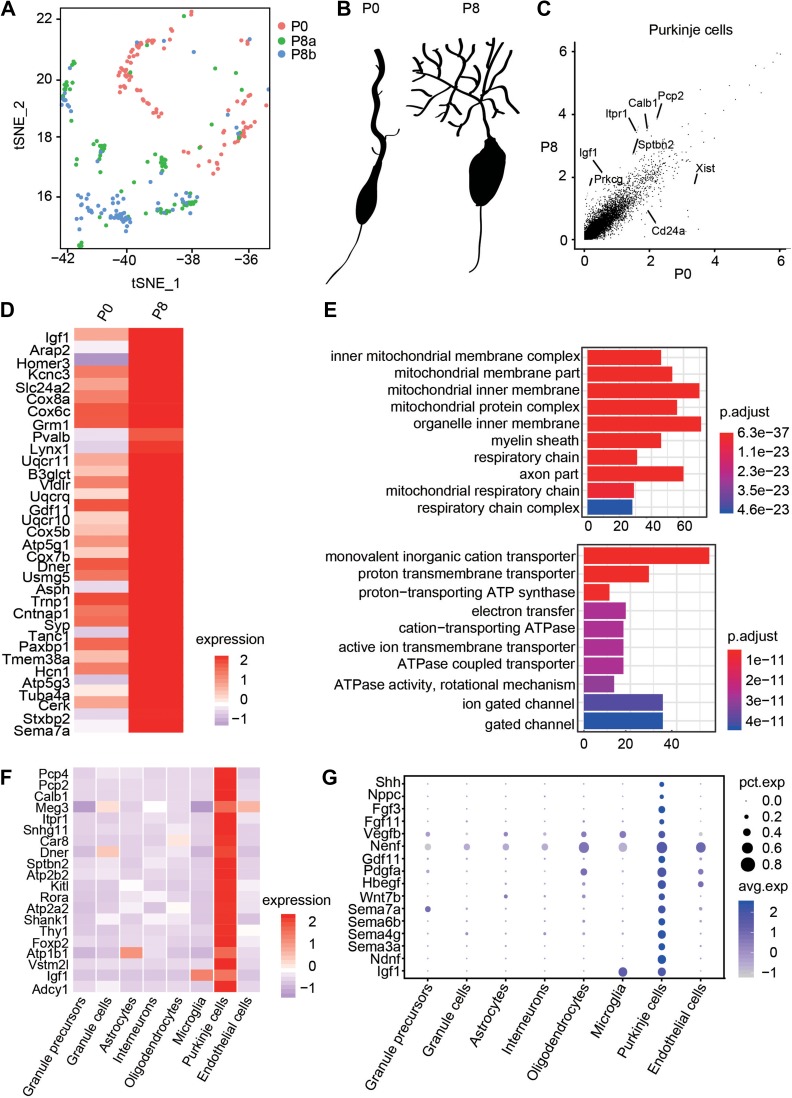
Molecular signature of PCs of developing mouse cerebellum. (**A**) t-SNE map showing a total of 256 PCs from one P0 sample and two P8 samples. (**B**) Schematic diagram describing the developmental process of PC morphology from P0 to P8. (**C**) Differential expression analysis for PCs between P0 and P8. (**D**) The heat-map showing a list of DEGs in PCs between P0 and P8. (**E**) The enriched GO terms of DEGs in PCs between P0 and P8. (**F**) The top 20 DEGs in PCs compared with other cell types. (**G**) Enriched expression of genes encoding secretory proteins in PCs compared with other cell types.

Dataset containing 256 PCs (including 92 from P0 and 164 from P8) was further analyzed for insights into molecular mechanisms underlying temporal development of PCs (Figure 2A). At P0, PCs usually exhibit bipolar morphology, named as simple-fusiform cells, followed by the development of planar and fan shaped dendrites (Figure 2B) (Sotelo and Rossi, 2013). This process is accompanied by proliferation of GPs and outside-in migration of differentiated GCs (Blazeski and Mason, 1994; Miyata et al., 1997; Rice and Curran, 1999). Although several molecules have been shown to be involved in PC morphogenesis (Kapfhammer, 2004; Fujishima et al., 2018), a complete transcriptome analysis is needed for deeper understanding about mechanisms of PC maturation and functions. We analyzed scRNA-seq dataset of individual PCs from P0 and P8 and identified 618 differentially expressed genes (DEGs) between these two time points (Supplementary Dataset S3). Strikingly, gene ontology (GO) analysis showed that many DEGs were involved in mitochondrial and ATPase biological processes (Figure 2C–E), suggesting that mitochondrial pathway may contribute to morphological development of PCs. Although PCs occupy a minority of cells in the cerebellum, they have critical non-autonomous roles in cerebellum development and circuit formation, including proliferation of GPs (Goldowitz and Hamre, 1998; Wechsler-Reya and Scott, 1999), migration of GCs (Rezai and Yoon, 1972), as well as synapse formation and refinement (Sassoe-Pognetto and Patrizi, 2017). We extensively analyzed the genes that were differentially expressed in PCs compared to other clusters using Seurat analysis, and identified 815 DEGs between PC and other cell types at P8 (Figure 2F and Supplementary Dataset S2). Notably, most of the top 20 divergent genes have been shown to play important roles in cerebellum development, such as genes encoding *insulin-like growth factor I* (*Igf1*), *nuclear receptor ROR-alpha* (*Rora*), and *inositol 1,4,5-trisphosphate receptor type 1* (*Itpr1*), which have been shown to be involved in PC morphogenesis (Hamilton et al., 1996; Fukudome et al., 2003; Van de Leemput et al., 2007). Considering non-autonomous roles of PCs, we analyzed DEGs encoding secretory proteins and identified 16 genes that were highly expressed in PCs (Figure 2G). Among them, several members of Semaphorin family of axon guidance proteins, including *Sema 3a*, *4 g*, *6b*, *7a*, *c-type natriuretic peptide* (*Nppc*), *neuron-derived neurotrophic factor* (*Ndnf*), and *platelet-derived growth factor subunit A* (*Pdgfa*), are highly expressed in subsets of PCs (Figure 2G). These results suggest potential regulators for neuronal morphogenesis, positioning, survival, or precise connections.

**Figure 3:**
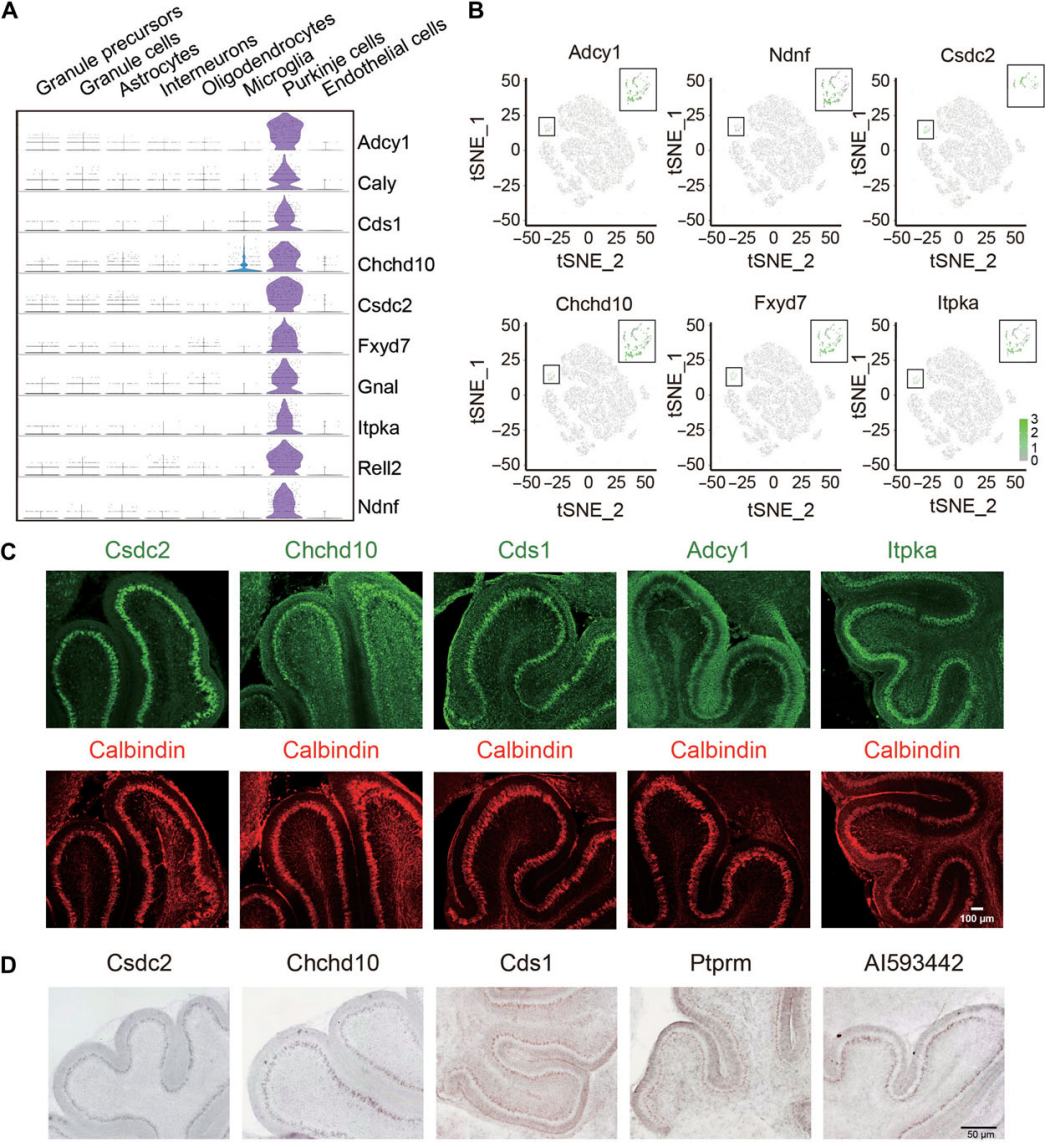
Identification of novel genes specifically expressed in PCs. (**A**) Violin plots showing the expression of novel genes in PCs. (**B**) t-SNE visualization of the expression of *Cds1, Csdc2, Chchd10, Ptprm, Itpka*, and *Adcy1* in PCs. (**C**) Immunostaining examination for the expression of indicated proteins in P8 cerebellum. Note their distribution in PC layer marked by Calbindin. Scale bar, 100 μm. (**D**) ISH analysis for the expression of target genes in the cerebellum of mice at P8. Scale bar, 50 μm.

In addition to the known marker genes of PC, such as Calbindin, we also found several other genes that were specifically expressed in PCs, including *cold shock domain-containing protein C2* (*Csdc2*), *coiled-coil-helix-coiled-coil-helix domain-containing 10* (*Chchd10*), *inositol-trisphosphate 3-kinase A* (*Itpka*), *phosphatidate cytidylyltransferase 1* (*Cds1*), and *adenylate cyclase type 1* (*Adcy1*) (Figure 3A and B). The expression of these genes in PCs was verified by immunostaining (Figure 3C) or *in situ* hybridization (ISH) (Figure 3D). Notably, Adcy1 protein was not only expressed in the soma of PCs, but also expressed in dendritic processes in outer external granule layer/molecular layer (Figure 3C). These results indicate novel marker genes expressed in PCs, whose functions remain to be investigated. Indeed, previous studies have shown that mutations of *Chchd10* are associated with several diseases, such as frontotemporal dementia and cerebellar ataxia (Bannwarth et al., 2014; Chaussenot et al., 2014). These findings refine and extend previous literatures about molecular specification of PCs and implicate mechanisms underlying cerebellar circuit formation.

### Expression pattern and co-expression network of ataxia risk genes

**Figure 4:**
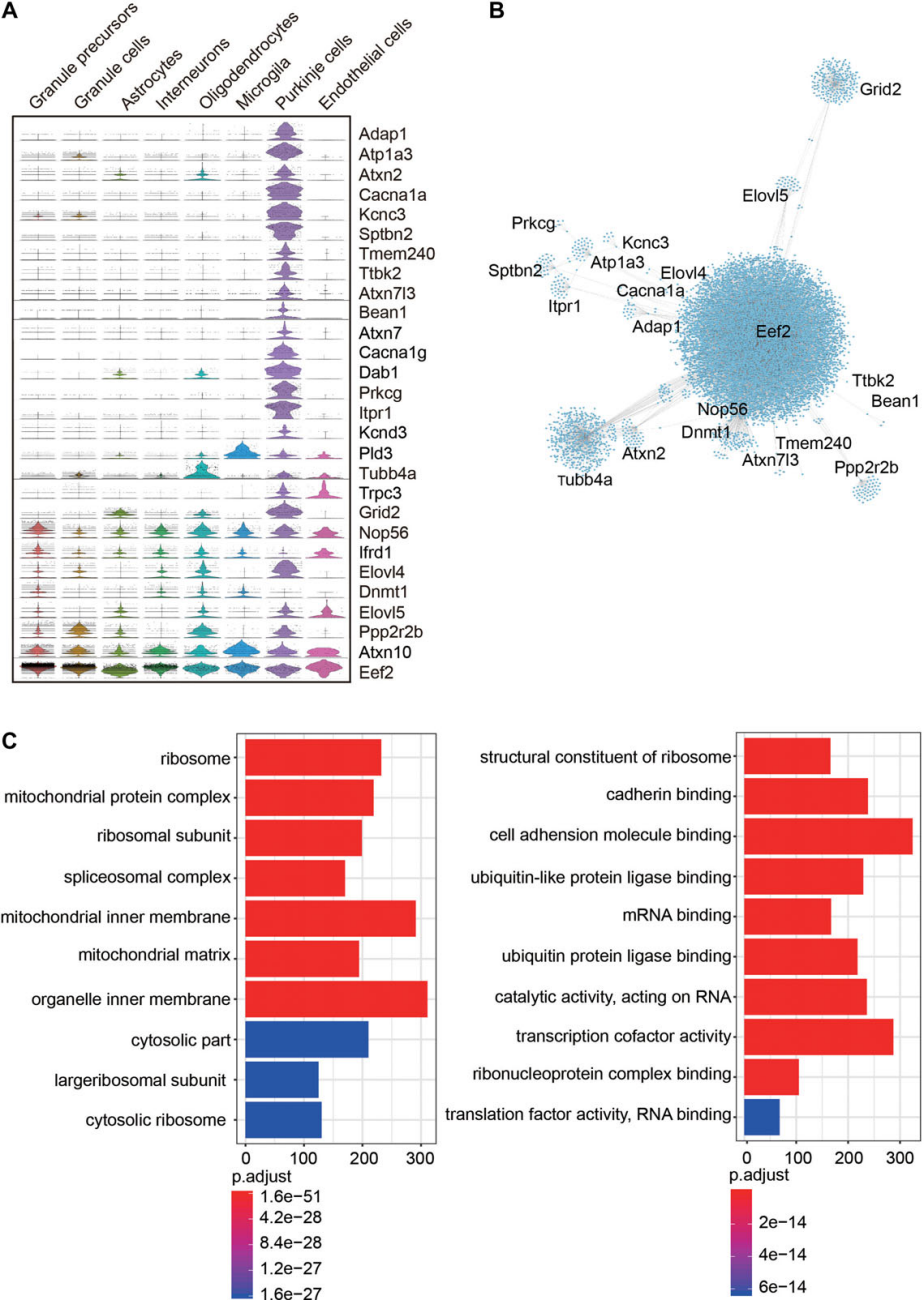
Expression patterns and coexpression networks of ataxia risk genes. (**A**) Violin plots showing the expression pattern of 28 ataxia risk genes in each neuron sub-cluster. (**B**) Gene coexpression network showing the genes coexpressed with ataxia risk genes. (**C**) Enriched biological processes for genes coexpressed with ataxia risk genes.

Previous studies have identified a number of ataxia risk genes (Paulson, 2009; Ashizawa et al., 2018), whereas susceptible cell types in the cerebellum remain unclear. We analyzed the expression pattern of 28 ataxia risk genes and found that majority of them are highly expressed in PCs, while only a few of them, including *elongation factor 2* (*Eef2*), *ataxin-10* (*Atxn10*), *nucleolar protein 56* (*Nop56*), are widely expressed in all cell types (Figure 4A). This finding is in line with the hypothesis that dysfunction of PCs is closely related with occurrence of ataxia disorder (Sakaguchi et al., 1996; Taroni and DiDonato, 2004; Walter et al., 2006). We also analyzed the co-expression network of those ataxia risk genes and revealed a central role of *Eef2*, *Grid2*, *Tubb4a* in the co-expression networks (Irrthum et al., 2010), while *Ttbk2* and *Bean1* have few interactions with other ataxia risk genes (Figure 4B). This result indicates that different ataxia risk genes may act through shared or diverse gene expression networks to regulate cerebellar development or functions. GO analysis of these co-expression genes showed that most significantly enriched terms were related with organizations of organelle and mitochondrial inner membrane (Figure 4C). Strikingly, GO enrichments of ataxia risk co-expression genes were largely consistent with DEGs of PCs between P0 and P8. Together, these results suggest that mitochondrial function is highly correlated with development of PCs and implicate cellular basis of motor behavior disorder.

### Gene regulation network for GC differentiation

**Figure 5:**
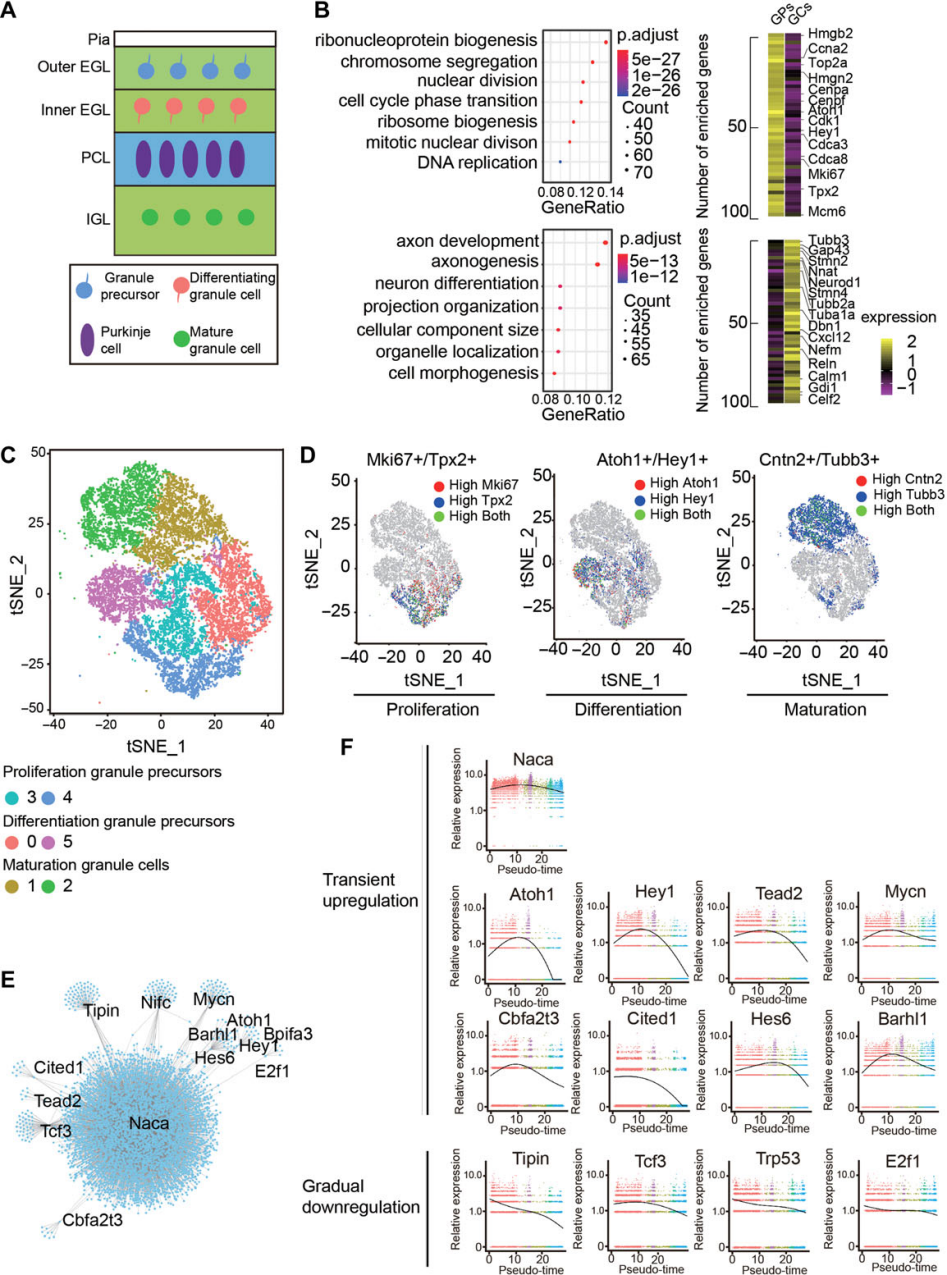
GC development and signaling pathways that regulate their maturation. (**A**) Schematic diagram representing the development of the GCs from proliferation, differentiation, to maturation. Pia, pial surface; EGL, external germinal layer; IGL, internal granular layer. (**B**) Differentially expressed genes between GPs and GCs and enriched biological processes for cells at each state. (**C**) t-SNE visualization of developing GCs at various states, ranging from proliferation, differentiation to maturation. (**D**) t-SNE visualization for the expression of proliferation-related genes *Mki67* and *Tpx2*, differentiation markers *Atoh1* and *Hey1*, and maturation-related markers *Cntn2* and *Tubb3*. (**E**) Transcription networks of the TFs regulating GC maturation. (**F**) Expression patterns of TFs related with GC development in pseudotime.

GCs account for around 80% of all neurons in the cerebellum, where they receive afferent inputs through 4–5 dendrites in the granule layer and form synapses with other cells in the molecular layer through the parallel fibers (Kalinichenko and Okhotin, 2005; Azevedo et al., 2009; Chédotal, 2010). GPs originated from the rhombic lip region migrate to cerebellar anlage and proliferate in the outer EGL, then exit the cell cycle to become migrating neuroblasts in the inner germinal layer. Differentiated GCs migrate into the IGL and mature around postnatal week 3 into fully functional excitatory GCs (Figure 5A). To reveal the developmental processes of GCs, we analyzed DEGs between GPs and GCs. The enriched GO terms in GPs were related to cell division and proliferation, while GCs enriched genes were related to axonal development and cell morphogenesis (Figure 5B). To investigate the developmental trajectory of GCs in more detail, a total of 17160 single GCs and GPs were further divided into 6 clusters with random forest algorithm (Figure 5C). Clusters 3 and 4 showed higher expression of proliferation genes, such as *Mki67* and *Tpx2* and thus represent proliferating GPs (Figure 5D). Clusters 0 and 5 represented newly differentiated GCs as they expressed high levels of *Atoh1* and *Hey1* (Figure 5D). Clusters 1 and 2 highly expressed neuronal maturation genes, such as *Cntn2* and *Tubb3*, and thus considered to be mature GCs (Figure 5D). To further explore the gene regulation network involved in GC differentiation, we selected top 13 TFs from DEGs of Cluster 0 and 5 and analyzed co-expression of these TFs with GC expression genes (Figure 5E). Notably, *nascent polypeptide-associated complex subunit alpha (Naca)*, a transcriptional co-activator which has been shown to be involved in developmental of cardiac and skeletal muscles (Park et al., 2010), exhibited the strongest co-regulation patterns with target genes, suggesting a potential role of *Naca* in regulating the differentiation of GCs (Figure 5E). To further display TFs that may orchestrate GCs differentiation, we reconstructed the developmental time-course using Monocle analysis (Trapnell et al., 2014) (Supplementary Figure S4A–D) and assessed the relative expression of 13 TFs in Pseudotime (Figure 5F) (Trapnell et al., 2014). We found that *Naca* showed persistent high-level expression in all stages, whereas differentiation-related genes, such as *Atoh1*, *Hey1*, *Barhl1* showed transient up-regulation in the intermediate stage, and proliferation-related genes, such as *Tipin* and *Cited*, exhibited gradual down-regulation (Figure 5F). These results demonstrate temporal and dynamic transcriptional program in the continuous process of GC trajectory.

### Implications of interneuron development and glia functions

**Figure 6:**
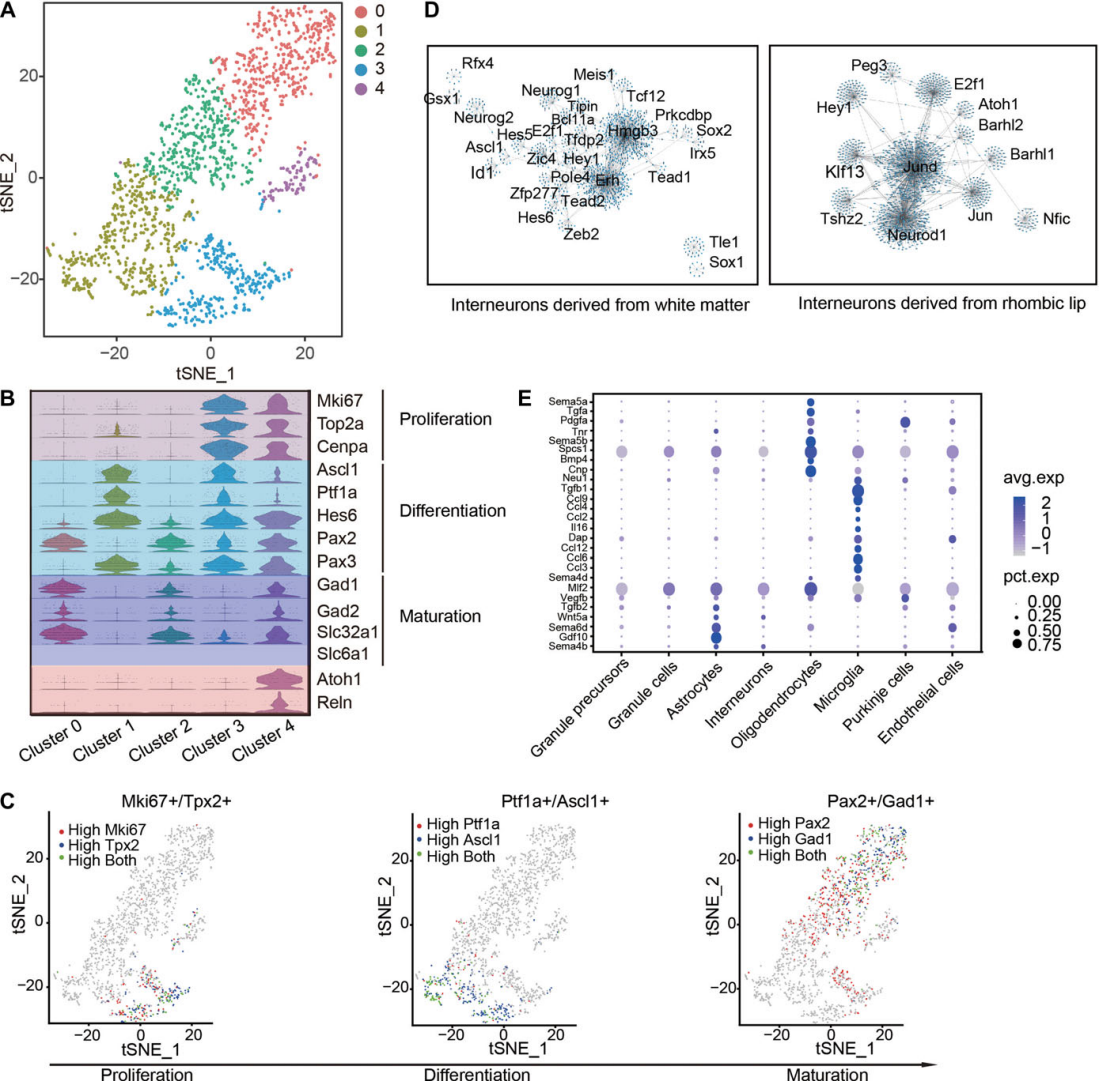
Transcriptional profiling of cerebellar interneurons and glia cells. (**A**) t-SNE map of interneuron linage cells. (**B**) Violin plots showing the expression patterns of genes associated with cell states and spatial origins. (**C**) t-SNE visualization of continous expression pattern of proliferation-related genes *Mki67* and *Tpx2*, differentiation-related genes *Ptf1a* and *Ascl1*, and maturation markers *Pax2* and *Gad1* during interneuron development. (**D**) Transcription networks of the TFs regulating differentiation of interneurons originated from cerebellar white matter (left) or rhombic lip (right). (**E**) Expression patterns of genes encoding secretory molecules in glia cells.

Cerebellum interneurons are a diverse population that varies widely in morphology, connectivity and patterns of activity. The basket/stellate cells in molecular layer and Golgi cells in granule layer are inhibitory interneurons derived from a common precursor population in the cerebellar white matter (Maricich and Herrup, 1999; Weisheit et al., 2006), whereas unipolar brush cells in granule layer are excitatory neurons derived from the rhombic lip (Englund et al., 2006). As indicated in Figure 6A, a total of 2034 interneurons or their progenitors were subdivided into 5 clusters. Among them, Clusters 1 and 3 represent inhibitory interneuron progenitors in proliferation or intermediate state, characterized by the expression of cerebellar inhibitory interneuron lineage marker genes *Ptf1a* and *Ascl1* without or with the expression of proliferation genes *Mki67* and *Top2a* (Figure 6B). The expression of *Pax3* in these cells agrees with a recent report that showed localization of *Pax3*-positive inhibitory neural precursors in the cerebellar white matter (Rosenberg et al., 2018). Clusters 0 and 2 represent differentiated inhibitory neurons, as reflected from the expression of *Gad1, Gad2*, and GABA transporter *Slc6a1* (Figure 6B and C). The expression of *Atoh1* and *Reelin* (*Reln*) in Cluster 4 suggested origin of this cell population from rhombic lip regions, and the expression of *Pax2* and *Slc32a1* defined this cluster as a lineage of inhibitory interneurons (Figure 6B). To further delineate gene regulatory network related with cerebellar interneuron differentiation, we extracted abundantly expressed TFs from Clusters 1 and 4, and analyzed co-expression network of those TFs, respectively. We found that *Erh* and *Hmgb3* are the candidate core TFs regulating the differentiation of Basket/Stellate/Golgi cells, and the distinct TF co-expression network in Cluster 4 suggests again a new subtype of inhibitory neuron linage derived from rhombic lip (Figure 6D).

It is known that glia cells play important roles in neural development (Mount et al., 1995; Marın-Teva et al., 2004; Zuchero and Barres, 2015), e.g. guiding nerve growth or neuronal migration, through non-autonomous mechanisms. However, the expression pattern of genes encoding secretory proteins in the developing cerebellum is not clear. From a total of 2826 glia cells (2034 astrocytes, 455 oligodendrocytes, 337 microglia) in our dataset, we identified 233 DEGs among microglia, astrocytes, and oligodendrocytes. Among them, several genes encoding Semaphorin family proteins were enriched in glial cells, e.g. *Sema6d* in astrocytes, *Sema5a* and *5b* in oligodendrocytes (Figure 6E). A series of genes encoding chemokine ligands were expressed in microglia, suggesting their roles in cerebellar development (Figure 6E).

## Discussion

Although mammalian cerebellum accounts for <10% of total brain mass and relatively few cell types, it contains more than half of neurons in the whole brain (Wang and Zoghbi, 2001; Lackey et al., 2018). Extensive efforts have been put to investigate development of the cerebellum, in particular factors that determine the generation, positioning, and differentiation of cerebellar neurons (Wechsler-Reya and Scott, 1999; Butts et al., 2014; Marzban et al., 2014). However, the dynamic gene expression networks that orchestrate cell fate trajectory and functions are not completely understood. Single-cell RNA sequencing provides a unprecedented angle for the discovery of new cell types, identifying specific marker genes for target cell types, understanding gene expression patterns specifying particular cell fate, and implicating molecular basis of cells to execute their functions (Pollen et al., 2015; Shin et al., 2015; Zeisel et al., 2015; La Manno et al., 2016; Marques et al., 2016). For instance, it has been widely used for the analysis of cell types in developing or adult cortex of various species, including mouse and human (Pollen et al., 2014; Zeisel et al., 2015; Telley et al., 2016; Nowakowski et al., 2017). In this study, we analyzed 21119 single cells from the cerebellum of postnatal mice. Different from most recent studies, which mainly validated cell types or provided a transcriptional atlas of the developing murine cerebellum (Carter et al., 2018; Rosenberg et al., 2018), our analysis focused on identifying molecules that are correlated with neuronal maturation and functions. Our analysis of ataxia-related genes implicates cell types that mediate abnormal processes in disease.

PCs are the most elaborate cell type in the whole brain with complex dendritic trees receiving around one hundred thousand synaptic inputs per cell (De Camilli et al., 1984). How is this particular morphology determined? Previous studies have shown the involvement of several factors in the morphogenesis of PCs, such as *Igf1*, *Rora*, *Itpr1*, *Foxp2*, and *Geranylgeranyltransferase I* (Fukudome et al., 2003; Boukhtouche et al., 2006; Wu et al., 2010; French et al., 2018). We identified 618 DEGs in PCs between P0 and P8 and found that most of them are involved in the mitochondria and ATPase pathways, suggesting roles of these biological processes in PC development and maturation. By analyzing DEGs among different cell clusters, we also identified novel genes specifically expressed in PCs, suggesting new markers of PCs. Because morphogenesis of murine PCs last several postnatal weeks (Berry and Bradley, 1976; Altman and Winfree, 1977), analysis for the gene expression profiles in later time points is necessary for more complete understanding of mechanisms underlying dendritic development, as well as synapse and circuitry formation.

Dysfunction of the cerebellar circuit causes abnormal behavioral outcomes, ranging from neurological to neuropsychiatric conditions, such as ataxia, tremor, and autism spectrum disorder (Lackey et al., 2018). A number of genes have been shown to be associated with cerebellum-associated diseases, but types of cells affected are mostly unclear. Here we analyzed the expression of 28 ataxia risk genes in each cerebellar cell type and the co-expression network of related genes. We found that around half of ataxia risk genes are associated with mitochondria pathway and, remarkably, specifically expressed in PCs. These results suggest cell types susceptible to mutations of disease risk genes. Functional validation of these genes in defined cell types using genetic approaches will further strengthen their functions in neuronal or circuitry development and provide deeper insights into cellular mechanisms of related diseases.

GCs in the cerebellum are the most numerous cell types in the brain. Their precursors are derived from upper rhombic lip at middle embryonic stage (E13.5 in mice), followed by continued proliferation after arriving at the EGL and sequential differentiation and migration at postnatal stages (Wang and Zoghbi, 2001). Thus, the cerebellum at P0 and P8 should contain GCs at all stages. Our scRNA-seq analysis indicates that this is indeed the case. We found continuous trajectory of GCs, ranging from proliferation, differentiation, to mature states. The expression dynamics of TFs in pseudo-time demonstrates a comprehensive genetic program governing state transitions. The expression of genes encoding neuronal cytoskeletal molecules in differentiated GCs suggests molecular mechanisms underlying morphological dynamics during migration.

We expect the results obtained from our dataset and analyses will expedite understanding of mechanisms of cerebellum development. The expression of disease-related genes in defined cell types will accelerate understanding of molecular and cellular mechanisms of cerebellar diseases.

## Materials and methods

### Animals

All animal usage and manipulations followed guidelines of Institutional Animal Care and Use Committees at the Institute of Neuroscience, Chinese Academy of Sciences. C57BL/6 J male mice at postnatal days 0 and 8 were used for single cell dissociation.

### Single-cell dissociation and 10x genomics chromium library construction

Dissection and cell dissociation were carried out as described previously (Wu et al., 2014). Briefly, entire cerebellum was separated from the whole brain using tweezers to remove the meninges. Then, the samples were sheared in 1×Hank’s balanced salt solution (HBSS) and digested for 15–20 min in 0.25% trypsin solution (Thermo Fisher Scientific). Dissociated cells were filtered through a 22-μm cell strainer (Millipore), pelleted by centrifugation at 300 g for 3 min, and re-suspended in a solution containing 0.04% BSA (Sigma) in 1× PBS (calcium and magnesium free) at a concentration of 2000–3000 cells/ml. Cell healthy state and numbers were measured by trypan blue staining. Dissociated cells in each sample were partitioned into nanoliter-scale Gel Bead-In-EMulsions (GEMs) in a limiting dilution to lower multiplet rate. Then, single-cell GEMs were incubated with reverse-transcription reagents, including several primers for cell and transcript barcoding in cDNA synthesis, followed by cDNA amplification and library construction. All procedures followed the guidelines provided by 10x Genomics, Inc.

### Single-cell RNA-seq data processing, filtering, and clustering

For RNA-seq data processing, we used the Cell Ranger software provided by 10x Genomics, which helps to convert 10x droplet data to matrices of expression counts. Briefly, the raw base call (BCL) files generated from the Illumina HiSeqX were demultiplexed into paired-end, gzip-compressed FASTQ files. Both pairs of FASTQ files were provided as input to ‘cellranger count’, and reads were aligned to mus musculus reference transcriptome (GenBank assembly accession: GCA_000001635.8). Both ‘cellranger mkfastq’ and ‘cellranger count’ were run with default command line options. Cells were first filtered to remove those that contained >6500 and <1000 genes detected. Genes that were detected in <6 cells were also removed. We next run canocical correlation analysis to indentify common sources of variation between samples and Multi-CCA to combine three objects to one object. Because we have begun to see drop-off in signal before CC15, we chose CC1–15 for subsequent analysis. The Seurat ‘FindClusters’ function was used to partition the cells into transcriptionally distinct clusters and present the graph clustering output on 2D map by t-SNE.

### Differentially expressed genes and GO analysis

The ‘FindAllMarkers’ function in Seurat was used to identify unique cluster-specific marker genes with threshold set as 0.25. The ‘roc’ test was used to evaluate ‘classification power’ for a certain gene (ranging from 0—random, to 1—perfect), and ‘avg.logFC’ represents fold change in the corresponding clusters. Violin plots, feature plot and heat maps were generated using Seurat. GO enrichment analysis was carried out by ‘ClusterProfiler’ in R software program.

### Developmental pseudotime analysis

The Monocle 2 package in R software program was used to determine the developmental pseudotime of GCs. Single-cell trajectories were constructed by DEGs based on the analysis of UMI reads data. Default settings were used for all other parameters. The regulation networks for the TFs were constructed by GENIE3 package and plotted by Cytoscape.

### Immunohistochemistry

C57BL/6 J male mice at P8 were perfused with 4% paraformaldehyde (PFA) in PBS and then the brains were dissected out and post-fixed in cold 4% PFA in PBS at 4°C overnight. The fixed brains were dehydrated in 30% sucrose in PBS at 4°C and cut into 30 μm cryosections collected on glass slides. Brain slices were washed in PBS for three times and permeated in antigen retrieval buffer for 5 min, followed by treatment with 0.3% (*v/v*) Triton X-100 in PBS for 25 min at room temperature (RT). Treated slices were incubated in a blocking solution (5% BSA in PBS) for 1 h, followed by incubation with primary antibodies at 4°C overnight, three washes with PBS, and then incubation with secondary antibodies. Primary antibodies used in immunohistochemistry include: rabbit anti-Itpka (Proteintech 14270-1-AP, 1:200); rabbit anti-Chchd10 (Proteintech 25671-1-AP, 1:200); rabbit anti-Adcy1 (Abcam ab69597, 1:250); rabbit anti-Csdc2 (Abcam ab221955, 1:500); rabbit anti-Cds1 (Novus Biologicals NBP1-33434, 1:100); and mouse anti-Calbindin (Sigma C9848, 1:1000). The fluorophore-conjugated secondary antibodies were from Life Technologies.

### In situ hybridization

Mice at P8 were perfused with RNase-inactivated PBS, followed by cold 4% PFA, and then the brains were post-fixed with 4% DEPC-treated PFA at 4°C overnight. The fixed brains were dehydrated in 20% sucrose (Sigma) in PBS at 4°C, then sectioned sagittally (30 μm thickness) with a cryostat (Leica, CM1950) and collected on superfrost plus microscope slides (Fisher Scientific). The probes complementary to target mouse mRNAs used for *in situ* hybridization (ISH) were cloned from mouse cDNAs into pGEMT plasmid using following primers: *CSDC2* forward 5′-GTGCTGCCCACTACAGGAGACAGT-3′; *CSDC2* reverse, 5′-TCAGAGGCCTGGCTTGTGAGAGGA-3′; *CHCHD10* forward, 5′-GTCTTGTGATGGGTCTCAGAGAACACAACC-3′; *CHCHD10* reverse 5′-ACCCCGCAGACTAACAGGAGACATAATTATTCAA-3′; *CDS1* forward 5′-ATGCTGGAGCTGCGGCAC-3′; *CDS1* reverse 5′-ATTACTCCCTCCCTCCATTCCTACAG-3′; *PTPRM* forward 5′-CAGCATCCTTTGCCAAACACCGTC-3′; *PTPRM* reverse 5′-GCCACCTCATAGCAGAACTTGTACTGATCC-3′; *AI593442* forward 5′-TCCATGCCTGCATCTGATATACAGCAAAGG-3′; *AI593442* reverse 5′-TTGGATGAGGGCAAGTTAAAGAGATTAATGG-3′. The anti-sense and sense strands were labeled by digoxin with DIG RNA Labeling Mix (Roche), followed by digestion of cDNA templates using DNase I. P8 mouse brain slices were incubated with synthesized anti-sense or sense RNA probes (1 μg/μl) at 70°C overnight, followed by incubation with alkaline phosphatase (AP)-labeled anti-digoxigenin Fab fragments (Roche, 1:2000) at 37°C for 16 h, followed by extensive washes. The sense and anti-sence probes were blindly used in ISH with all sense probes giving negative signal. Images were collected with Olympus VS120.

### Data availability

The raw data reported in this work were deposited to NCBI with the accession numbers GEO: GSE122357 or SRP165255.

## Supplementary Material

suppl

## References

[mjy089C1] Adams N.C., Tomoda T., Cooper M., et al. (2002). Mice that lack astrotactin have slowed neuronal migration. Development 129, 965–972. 11861479 10.1242/dev.129.4.965

[mjy089C2] Altman J., and Bayer S.A. (1997). Development of the Cerebellar System: In Relation to Its Evolution, Structure, and Functions (1st edn). Boca Raton, Florida: CRC Press.

[mjy089C3] Altman J., and Winfree A.T. (1977). Postnatal development of the cerebellar cortex in the rat: V. Spatial organization of Purkinje cell perikarya. J. Comp. Neurol. 171, 1–16. 830668 10.1002/cne.901710102

[mjy089C4] Ashizawa T., Oz G., and Paulson H.L. (2018). Spinocerebellar ataxias: prospects and challenges for therapy development. Nat. Rev. Neurol. 14, 590–605. 30131520 10.1038/s41582-018-0051-6 PMC6469934

[mjy089C5] Azevedo F.A.C., Carvalho L.R.B., Grinberg L.T., et al. (2009). Equal numbers of neuronal and nonneuronal cells make the human brain an isometrically scaled-up primate brain. J. Comp. Neurol. 513, 532–541. 19226510 10.1002/cne.21974

[mjy089C6] Bannwarth S., Ait-El-Mkadem S., Chaussenot A., et al. (2014). A mitochondrial origin for frontotemporal dementia and amyotrophic lateral sclerosis through CHCHD10 involvement. Brain 137, 2329–2345. 24934289 10.1093/brain/awu138 PMC4107737

[mjy089C7] Bastian A.J. (2011). Moving, sensing and learning with cerebellar damage. Curr. Opin. Neurobiol. 21, 596–601. 21733673 10.1016/j.conb.2011.06.007 PMC3177958

[mjy089C8] Baumann O., Borra R.J., Bower J.M., et al. (2015). Consensus paper: the role of the cerebellum in perceptual processes. Cerebellum 14, 197–220. 25479821 10.1007/s12311-014-0627-7 PMC4346664

[mjy089C9] Ben-Arie N., Bellen H.J., Armstrong D.L., et al. (1997). Math1 is essential for genesis of cerebellar granule neurons. Nature 390, 169–172. 9367153 10.1038/36579

[mjy089C10] Berry M., and Bradley P. (1976). The growth of the dendritic trees of Purkinje cells in irradiated agranular cerebellar cortex. Brain Res. 116, 361–387. 974782 10.1016/0006-8993(76)90487-x

[mjy089C11] Blazeski R., and Mason C.A. (1994). Cell-cell interactions influence survival and differentiation of purified Purkinje cells in vitro. Neuron 12, 243–260. 8110456 10.1016/0896-6273(94)90268-2

[mjy089C12] Boukhtouche F., Janmaat S., Vodjdani G., et al. (2006). Retinoid-related orphan receptor α controls the early steps of Purkinje cell dendritic differentiation. J. Neurosci. 26, 1531–1538. 16452676 10.1523/JNEUROSCI.4636-05.2006 PMC6675487

[mjy089C13] Buckner R.L. (2013). The cerebellum and cognitive function: 25 years of insight from anatomy and neuroimaging. Neuron 80, 807–815. 24183029 10.1016/j.neuron.2013.10.044

[mjy089C14] Butler A., Hoffman P., Smibert P., et al. (2018). Integrating single-cell transcriptomic data across different conditions, technologies, and species. Nat. Biotechnol. 36, 411–420. 29608179 10.1038/nbt.4096 PMC6700744

[mjy089C15] Butts T., Green M.J., and Wingate R.J. (2014). Development of the cerebellum: simple steps to make a ‘little brain’. Development 141, 4031–4041. 25336734 10.1242/dev.106559

[mjy089C16] Caligiore D., Pezzulo G., Baldassarre G., et al. (2017). Consensus paper: towards a systems-level view of cerebellar function: the interplay between cerebellum, basal ganglia, and cortex. Cerebellum 16, 203–229. 26873754 10.1007/s12311-016-0763-3 PMC5243918

[mjy089C17] Carter R.A., Bihannic L., Rosencrance C., et al. (2018). A single-cell transcriptional atlas of the developing murine cerebellum. Curr. Biol. 28, 2910–2920. 30220501 10.1016/j.cub.2018.07.062

[mjy089C18] Chaussenot A., Le Ber I., Ait-El-Mkadem S., et al. (2014). Screening of CHCHD10 in a French cohort confirms the involvement of this gene in frontotemporal dementia with amyotrophic lateral sclerosis patients. Neurobiol. Aging 35, 1–4. 25155093 10.1016/j.neurobiolaging.2014.07.022

[mjy089C19] Chédotal A. (2010). Should I stay or should I go? Becoming a granule cell. Trends Neurosci. 33, 163–172. 20138673 10.1016/j.tins.2010.01.004

[mjy089C20] De Camilli P., Miller P., Levitt P., et al. (1984). Anatomy of cerebellar Purkinje cells in the rat determined by a specific immunohistochemical marker. Neuroscience 11, 761–817. 6330609 10.1016/0306-4522(84)90193-3

[mjy089C21] D’Angelo E., and Casali S. (2012). Seeking a unified framework for cerebellar function and dysfunction: from circuit operations to cognition. Front. Neural. Circuits 6, 1–23. 23335884 10.3389/fncir.2012.00116 PMC3541516

[mjy089C22] Englund C., Kowalczyk T., Daza R.A., et al. (2006). Unipolar brush cells of the cerebellum are produced in the rhombic lip and migrate through developing white matter. J. Neurosci. 26, 9184–9195. 16957075 10.1523/JNEUROSCI.1610-06.2006 PMC6674506

[mjy089C23] Fleming J.T., He W., Hao C., et al. (2013). The Purkinje neuron acts as a central regulator of spatially and functionally distinct cerebellar precursors. Dev. Cell 27, 278–292. 24229643 10.1016/j.devcel.2013.10.008 PMC3860749

[mjy089C24] French C.A., Vinueza Veloz M.F., Zhou K., et al. (2018). Differential effects of Foxp2 disruption in distinct motor circuits. Mol. Psychiatry10.1038/s41380-018-0199-x. PMC6514880 30108312

[mjy089C25] Fujishima K., Kawabata Galbraith K., and Kengaku M. (2018). Dendritic self-avoidance and morphological development of cerebellar purkinje cells. Cerebellum 17, 701–708. 30270408 10.1007/s12311-018-0984-8

[mjy089C26] Fukudome Y., Tabata T., Miyoshi T., et al. (2003). Insulin-like growth factor-I as a promoting factor for cerebellar Purkinje cell development. Eur. J. Neurosci. 17, 2006–2016. 12786966 10.1046/j.1460-9568.2003.02640.x

[mjy089C27] Glickstein M., Strata P., and Voogd J. (2009). Cerebellum: history. Neuroscience 162, 549–559. 19272426 10.1016/j.neuroscience.2009.02.054

[mjy089C28] Goldowitz D., and Hamre K. (1998). The cells and molecules that make a cerebellum. Trends Neurosci. 21, 375–382. 9735945 10.1016/s0166-2236(98)01313-7

[mjy089C29] Gupta I., Collier P.G., Haase B., et al. (2018). Single-cell isoform RNA sequencing characterizes isoforms in thousands of cerebellar cells. Nat. Biotechnol. 36, 1197–1205. 10.1038/nbt.4259 30320766

[mjy089C30] Hamilton B.A., Frankel W.N., Kerrebrock A.W., et al. (1996). Disruption of the nuclear hormone receptor RORα in staggerer mice. Nature 379, 736–739. 8602221 10.1038/379736a0

[mjy089C31] Hatten M.E., and Heintz N. (1995). Mechanisms of neural patterning and specification in the development cerebellum. Annu. Rev. Neurosci. 18, 385–408. 7605067 10.1146/annurev.ne.18.030195.002125

[mjy089C32] Hoshino M. (2006). Molecular machinery governing GABAergic neuron specification in the cerebellum. Cerebellum 5, 193–198. 16997750 10.1080/14734220600589202

[mjy089C33] Irrthum A., Wehenkel L., and Geurts P. (2010). Inferring regulatory networks from expression data using tree-based methods. PLoS One 5, 1–10. 10.1371/journal.pone.0012776 PMC2946910 20927193

[mjy089C34] Kalinichenko S., and Okhotin V. (2005). Unipolar brush cells–a new type of excitatory interneuron in the cerebellar cortex and cochlear nuclei of the brainstem. Neurosci. Behav. Physiol. 35, 21–36. 15739785 10.1023/b:neab.0000049648.20702.ad

[mjy089C35] Kapfhammer J.P. (2004). Cellular and molecular control of dendritic growth and development of cerebellar Purkinje cells. Prog. Histochem. Cytochem. 39, 131–182. 15580762 10.1016/j.proghi.2004.07.002

[mjy089C36] Komuro H., and Yacubova E. (2003). Recent advances in cerebellar granule cell migration. Cell. Mol. Life Sci. 60, 1084–1098. 12861377 10.1007/s00018-003-2248-z PMC11138937

[mjy089C37] Koziol L.F., Budding D., Andreasen N., et al. (2014). Consensus paper: the cerebellum’s role in movement and cognition. Cerebellum 13, 151–177. 23996631 10.1007/s12311-013-0511-x PMC4089997

[mjy089C38] La Manno G., Gyllborg D., Codeluppi S., et al. (2016). Molecular diversity of midbrain development in mouse, human, and stem cells. Cell 167, 566–580. 27716510 10.1016/j.cell.2016.09.027 PMC5055122

[mjy089C39] Lackey E.P., Heck D.H., and Sillitoe R.V. (2018). Recent advances in understanding the mechanisms of cerebellar granule cell development and function and their contribution to behavior. F1000Res 7, 1–12. 10.12688/f1000research.15021.1 PMC6069759 30109024

[mjy089C40] Lang E.J., Apps R., Bengtsson F., et al. (2017). The roles of the olivocerebellar pathway in motor learning and motor control. A consensus paper. Cerebellum 16, 230–252. 27193702 10.1007/s12311-016-0787-8 PMC5116294

[mjy089C41] Macosko E.Z., Basu A., Satija R., et al. (2015). Highly parallel genome-wide expression profiling of individual cells using nanoliter droplets. Cell 161, 1202–1214. 26000488 10.1016/j.cell.2015.05.002 PMC4481139

[mjy089C42] Maricich S.M., and Herrup K. (1999). Pax-2 expression defines a subset of GABAergic interneurons and their precursors in the developing murine cerebellum. J. Neurobiol. 41, 281–294. 10512984 10.1002/(sici)1097-4695(19991105)41:2<281::aid-neu10>3.0.co;2-5

[mjy089C43] Marques S., Zeisel A., Codeluppi S., et al. (2016). Oligodendrocyte heterogeneity in the mouse juvenile and adult central nervous system. Science 352, 1326–1329. 27284195 10.1126/science.aaf6463 PMC5221728

[mjy089C44] Marzban H., Del Bigio M.R., Alizadeh J., et al. (2014). Cellular commitment in the developing cerebellum. Front. Cell. Neurosci. 8, 1–26. 25628535 10.3389/fncel.2014.00450 PMC4290586

[mjy089C45] Marın-Teva J.L., Dusart I., Colin C., et al. (2004). Microglia promote the death of developing Purkinje cells. Neuron 41, 535–547. 14980203 10.1016/s0896-6273(04)00069-8

[mjy089C46] Miyata T., Nakajima K., Mikoshiba K., et al. (1997). Regulation of Purkinje cell alignment by reelin as revealed with CR-50 antibody. J. Neurosci. 17, 3599–3609. 9133383 10.1523/JNEUROSCI.17-10-03599.1997 PMC6573700

[mjy089C47] Mount H., Dean D.O., Alberch J., et al. (1995). Glial cell line-derived neurotrophic factor promotes the survival and morphologic differentiation of Purkinje cells. Proc. Natl Acad. Sci. USA 92, 9092–9096. 7568079 10.1073/pnas.92.20.9092 PMC40930

[mjy089C48] Nowakowski T.J., Bhaduri A., Pollen A.A., et al. (2017). Spatiotemporal gene expression trajectories reveal developmental hierarchies of the human cortex. Science 358, 1318–1323. 29217575 10.1126/science.aap8809 PMC5991609

[mjy089C49] Park C.Y., Pierce S.A., von Drehle M., et al. (2010). skNAC, a Smyd1-interacting transcription factor, is involved in cardiac development and skeletal muscle growth and regeneration. Proc. Natl Acad. Sci. USA 107, 20750–20755. 21071677 10.1073/pnas.1013493107 PMC2996447

[mjy089C50] Paulson H.L. (2009). The spinocerebellar ataxias. J. Neuroophthalmol. 29, 227–237. 19726947 10.1097/WNO0b013e3181b416de PMC2739122

[mjy089C51] Pollen A.A., Nowakowski T.J., Chen J., et al. (2015). Molecular identity of human outer radial glia during cortical development. Cell 163, 55–67. 26406371 10.1016/j.cell.2015.09.004 PMC4583716

[mjy089C52] Pollen A.A., Nowakowski T.J., Shuga J., et al. (2014). Low-coverage single-cell mRNA sequencing reveals cellular heterogeneity and activated signaling pathways in developing cerebral cortex. Nat. Biotechnol. 32, 1–9. 25086649 10.1038/nbt.2967 PMC4191988

[mjy089C53] Rakic P. (1971). Neuron-glia relationship during granule cell migration in developing cerebellar cortex. A Golgi and electronmicroscopic study in Macacus Rhesus. J. Comp. Neurol. 141, 283–312. 4101340 10.1002/cne.901410303

[mjy089C55] Reeber S.L., Otis T.S., and Sillitoe R.V. (2013). New roles for the cerebellum in health and disease. Front. Syst. Neurosci. 7, 1–11. 24294192 10.3389/fnsys.2013.00083 PMC3827539

[mjy089C56] Rezai Z., and Yoon C.H. (1972). Abnormal rate of granule cell migration in the cerebellum of ‘weaver’ mutant mice. Dev. Biol. 29, 17–26. 4561458 10.1016/0012-1606(72)90039-5

[mjy089C57] Rice D.S., and Curran T. (1999). Mutant mice with scrambled brains: understanding the signaling pathways that control cell positioning in the CNS. Genes Dev. 13, 2758–2773. 10557205 10.1101/gad.13.21.2758

[mjy089C58] Rosenberg A.B., Roco C.M., Muscat R.A., et al. (2018). Single-cell profiling of the developing mouse brain and spinal cord with split-pool barcoding. Science 360, 176–182. 29545511 10.1126/science.aam8999 PMC7643870

[mjy089C59] Sakaguchi S., Katamine S., Nishida N., et al. (1996). Loss of cerebellar Purkinje cells in aged mice homozygous for a disrupted PrP gene. Nature 380, 528–531. 8606772 10.1038/380528a0

[mjy089C60] Sassoe-Pognetto M., and Patrizi A. (2017). The Purkinje cell as a model of synaptogenesis and synaptic specificity. Brain Res. Bull. 129, 12–17. 27721030 10.1016/j.brainresbull.2016.10.004

[mjy089C61] Satija R., Farrell J.A., Gennert D., et al. (2015). Spatial reconstruction of single-cell gene expression data. Nat. Biotechnol. 33, 495–502. 25867923 10.1038/nbt.3192 PMC4430369

[mjy089C62] Shin J., Berg D.A., Zhu Y., et al. (2015). Single-cell RNA-Seq with waterfall reveals molecular cascades underlying adult neurogenesis. Cell Stem Cell 17, 1–13. 26299571 10.1016/j.stem.2015.07.013 PMC8638014

[mjy089C63] Sotelo C., and Rossi F. (2013). Purkinje cell migration and differentiation In: Manto M., Schmahmann J.D., Rossi F., et al. (eds) Handbook of the Cerebellum and Cerebellar Disorders. Dordrecht: Springer, 147–178.

[mjy089C64] Taroni F., and DiDonato S. (2004). Pathways to motor incoordination: the inherited ataxias. Nat. Rev. Neurosci. 5, 641–655. 15263894 10.1038/nrn1474

[mjy089C65] Telley L., Govindan S., Prados J., et al. (2016). Sequential transcriptional waves direct the differentiation of newborn neurons in the mouse neocortex. Science 351, 1443–1446. 26940868 10.1126/science.aad8361

[mjy089C66] Trapnell C., Cacchiarelli D., Grimsby J., et al. (2014). The dynamics and regulators of cell fate decisions are revealed by pseudotemporal ordering of single cells. Nat. Biotechnol. 32, 381–386. 24658644 10.1038/nbt.2859 PMC4122333

[mjy089C67] Van de Leemput J., Chandran J., Knight M.A., et al. (2007). Deletion at ITPR1 underlies ataxia in mice and spinocerebellar ataxia 15 in humans. PLoS Genet. 3, 1076–1082. 10.1371/journal.pgen.0030108 PMC1892049 17590087

[mjy089C68] Walter J.T., Alvina K., Womack M.D., et al. (2006). Decreases in the precision of Purkinje cell pacemaking cause cerebellar dysfunction and ataxia. Nat. Neurosci. 9, 389–397. 16474392 10.1038/nn1648

[mjy089C69] Wang V.Y., and Zoghbi H.Y. (2001). Genetic regulation of cerebellar development. Nat. Rev. Neurosci. 2, 484. 11433373 10.1038/35081558

[mjy089C70] Wechsler-Reya R.J., and Scott M.P. (1999). Control of neuronal precursor proliferation in the cerebellum by Sonic Hedgehog. Neuron 22, 103–114. 10027293 10.1016/s0896-6273(00)80682-0

[mjy089C71] Weisheit G., Gliem M., Endl E., et al. (2006). Postnatal development of the murine cerebellar cortex: formation and early dispersal of basket, stellate and Golgi neurons. Eur. J. Neurosci. 24, 466–478. 16903854 10.1111/j.1460-9568.2006.04915.x

[mjy089C72] Weyer A., and Schilling K. (2003). Developmental and cell type-specific expression of the neuronal marker NeuN in the murine cerebellum. J. Neurosci. Res. 73, 400–409. 12868073 10.1002/jnr.10655

[mjy089C73] Wu K.-Y., He M., Hou Q.-Q., et al. (2014). Semaphorin 3A activates the guanosine triphosphatase Rab5 to promote growth cone collapse and organize callosal axon projections. Sci. Signal. 7, 1–13. 10.1126/scisignal.2005334 PMC4659511 25161316

[mjy089C74] Wu K.Y., Zhou X.P., and Luo Z.G. (2010). Geranylgeranyltransferase I is essential for dendritic development of cerebellar Purkinje cells. Mol. Brain 3, 1–12. 20540740 10.1186/1756-6606-3-18 PMC2902468

[mjy089C75] Zeisel A., Muñoz-Manchado A.B., Codeluppi S., et al. (2015). Cell types in the mouse cortex and hippocampus revealed by single-cell RNA-seq. Science 347, 1138–1142. 25700174 10.1126/science.aaa1934

[mjy089C76] Zilionis R., Nainys J., Veres A., et al. (2017). Single-cell barcoding and sequencing using droplet microfluidics. Nat. Protoc. 12, 44–73. 27929523 10.1038/nprot.2016.154

[mjy089C77] Zuchero J.B., and Barres B.A. (2015). Glia in mammalian development and disease. Development 142, 3805–3809. 26577203 10.1242/dev.129304 PMC4712885

