# Supplementary material for: Single-cell transcriptomes reveal molecular specializations of neuronal cell types in the developing cerebellum
Source: J Mol Cell Biol. 2019 Jan 25;11(8):636–48. doi: 10.1093/jmcb/mjy089 (PMC6788728; doi:10.1093/jmcb/mjy089)
Supplement: suppl [file suppl.zip › mjy089_Supplementary_Information-20181218final.doc]

# Figure S1. Quality control of scRNA-seq datasets. (A) Distribution of number of genes (nGene)，UMI counts (nUMI), and percentage of mitochondrial transcript reads counts among total reads counts (percent.mito) in each cell from samples at P0 and P8. (B) Negative correlation between percentage of mitochondrial genes and numbers of UMIs in each sample. (C) Positive correlation between number of genes and UMI counts in each sample. (D) Correlation between shared correlation strength and CC for three samples.

# Figure S2. Cell type identification in the mouse cerebellum. (A) Heat map showing the expression of marker genes in eight major cell types. (B) t-SNE map for the relative expression of known marker genes in indicated cell types. PCs: Purkinje cells; GPs: granule precursors; GCs: granule cells; ECs: endothelial cells. (C) Expression of Bergmann glia marker genes in astrocytes.

# Figure S3. Distribution of number of genes (nGene) (A)，UMI counts (nUMI) (B), and percentage of mitochondrial transcript reads counts among total reads counts (percent.mito) (C) in each cell type.

# Figure S4. Relative expression of known marker genes during granule cell development. (A, B) Analysis of granule cell trajectory by Monocle 2. (C) Branched heat map showing gene expression profiling of developing granule cells at various states in pseudotime. (D) Expression of representative genes during granule cell development in pseudo-time.
